# Expression of periaxin (PRX) specifically in the human cerebrovascular system: PDZ domain-mediated strengthening of endothelial barrier function

**DOI:** 10.1038/s41598-018-28190-7

**Published:** 2018-07-03

**Authors:** Michael M. Wang, Xiaojie Zhang, Soo Jung Lee, Snehaa Maripudi, Richard F. Keep, Allison M. Johnson, Svetlana M. Stamatovic, Anuska V. Andjelkovic

**Affiliations:** 1 Department of Neurology, University of Michigan, Ann Arbor, MI 48109 USA; 2 Department of Molecular and Integrative Physiology, University of Michigan, Ann Arbor, MI 48109 USA; 3 Neurology Service, VA Ann Arbor Healthcare System, Department of Veterans Affairs, Ann Arbor, MI 48105 USA; 4 Neuroscience Program, University of Michigan, Ann Arbor, MI 48109 USA; 5 Department of Neurosurgery, University of Michigan, Ann Arbor, MI 48109 USA; 6 Molecular and Cellular Pathology Graduate Programs, University of Michigan, Ann Arbor, MI 48109 USA; 7 Department of Pathology, University of Michigan, Ann Arbor, MI 48109 USA

## Abstract

Regulation of cerebral endothelial cell function plays an essential role in changes in blood-brain barrier permeability. Proteins that are important for establishment of endothelial tight junctions have emerged as critical molecules, and PDZ domain containing-molecules are among the most important. We have discovered that the PDZ-domain containing protein periaxin (PRX) is expressed in human cerebral endothelial cells. Surprisingly, PRX protein is not detected in brain endothelium in other mammalian species, suggesting that it could confer human-specific vascular properties. In endothelial cells, PRX is predominantly localized to the nucleus and not tight junctions. Transcriptome analysis shows that PRX expression suppresses, by at least 50%, a panel of inflammatory markers, of which 70% are Type I interferon response genes; only four genes were significantly activated by PRX expression. When expressed in mouse endothelial cells, PRX strengthens barrier function, significantly increases transendothelial electrical resistance (~35%; p < 0.05), and reduces the permeability of a wide range of molecules. The PDZ domain of PRX is necessary and sufficient for its barrier enhancing properties, since a splice variant (S-PRX) that contains only the PDZ domain, also increases barrier function. PRX also attenuates the permeability enhancing effects of lipopolysaccharide. Collectively, these studies suggest that PRX could potentially regulate endothelial homeostasis in human cerebral endothelial cells by modulating inflammatory gene programs.

## Introduction

Human cerebral endothelial cells possess special properties that are integral for normal homeostatic brain function. In particular, endothelial cells participate in the formation of the blood-brain barrier (BBB), a key function of the cerebrovasculature that prevents transit of blood-borne substances into the central nervous system^1–3^. A defined set of endothelial proteins is critical for the establishment and maintenance of this barrier function^4^. One important group of proteins includes transmembrane tight junction molecules (including claudin5^5^, occludin^6^, and junctional adhesion molecule^7^). Another key group of proteins includes cytoplasmic adaptor proteins that localize with tight junction membrane proteins. Important cerebral endothelial adaptor proteins described to date include the MAGUK class of proteins (ZO1^8^, ZO2^9^, and ZO3^10^). Additional proteins in endothelial barriers include cingulin^11^, AF-6^12^, and 7H6^13^.

Each of these adapter proteins contains a homologous and evolutionarily conserved structural motif, the PDZ domain (**p**ostsynaptic density protein of 95 kilodaltons, **d**isc large, **z**ona occludens-1). This domain, described in several membrane associated scaffolding molecules, binds to the carboxy-terminal tail of many of the tight junction transmembrane proteins^14–17^. The presence of multiple PDZ domains and other protein binding motifs in these endothelial adapter proteins mediates molecular linkage between junctional membrane proteins to the underlying actin cytoskeleton networks within the cell^18,19^. These adaptor proteins play an important role in regulating barrier properties, including in disease states^4^.

The multitude of PDZ-domain containing proteins that regulate endothelial cell function suggests there may be additional regulatory proteins containing similar domains. To identify new human brain endothelial cell proteins, we screened a public database for cerebral factors with vascular expression. From this screen, we identified periaxin (PRX) as a novel human cerebral endothelial PDZ-domain protein. Unlike in humans, PRX was not expressed in the cerebral endothelium in multiple other species, even though it was present in other tissues. Expression of PRX in cerebral endothelial cells *in vitro* tightened the BBB and altered inflammatory responses. However, PRX was primarily present in the nucleus rather than associated with tight junctions at the cell membrane, suggesting the effects of PRX, while PDZ dependent, are mediated by regulating gene expression.

## Results

### Identification of a human specific PDZ-domain protein in cerebral endothelial cells

**Figure 1:**
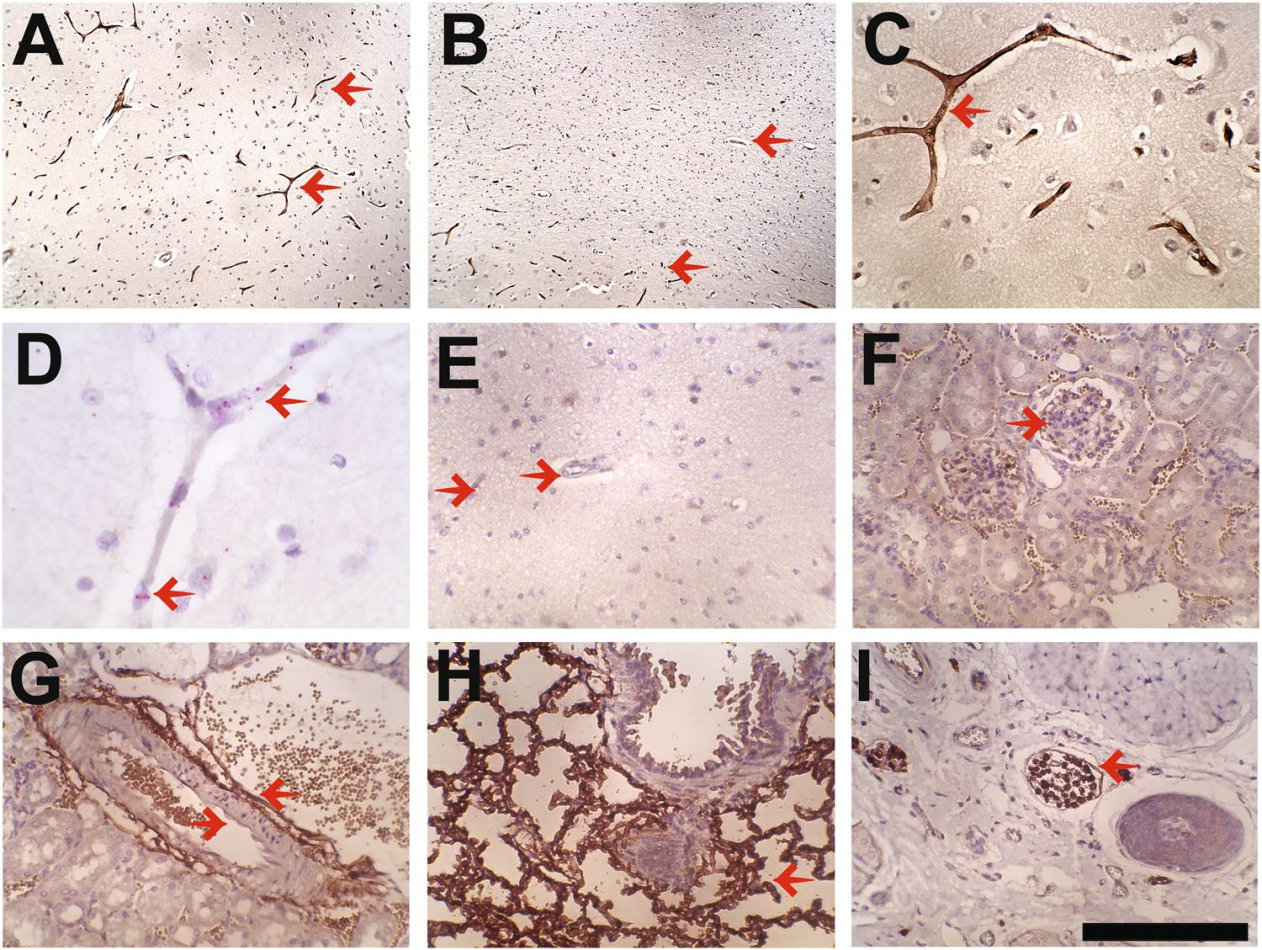
Expression of PRX in the human brain and in non-human tissues. Immunohistochemical analysis of human brain was performed with antibodies raised against human PRX. In the cerebral cortex, capillary staining (but not neurons or astrocytes) was detected in both gray (**A**) and white (**B**) matter. High power images demonstrate capillary and small vessel endothelial-predominant expression in the cortex (**C**). Positive vascular signals are highlighted with left pointing arrows. PRX mRNA was localized to capillaries by *in situ* hybridization ((**D**) pink granular stain shows RNA hybridization; left pointing arrows). Immunohistochemical analysis of PRX in mouse cortex (**E**) kidney (**F**,**G**) lung (**H**) and tail (**I**) was conducted for PRX expression using the same antibodies. Kidney and tail tissues demonstrated peripheral nerve staining while lung showed capillary expression (left pointing arrows). Right pointing arrows in (**E**–**G**) show endothelial cells that fail to stain for PRX in brain and kidney. Negative staining was observed in rat, pig, and marmoset brain (not shown). Scale bar for (**A**,**B**) shows 600 microns and for all other photographs measures 150 microns.

While conducting a bioinformatic study of protein expression patterns in the brain^20^, we identified PRX as a previously unrecognized brain endothelial protein in humans based on images from the Human Protein Atlas. To validate the expression pattern in an independent set of human samples, we immunostained for PRX in frontal cortex obtained at autopsy from 8 individuals (Fig. 1). Our staining for PRX showed strong capillary expression without neuronal or astrocyte reactivity (Fig. 1A and C). In addition, the endothelium of small white matter vessels were stained (Fig. 1B). Large arteries and veins of the leptomeninges did not react with PRX antibodies. *In situ* hybridization localized PRX mRNA to endothelial cells of the cortex, in agreement with immunohistochemistry results (Fig. 1D).

PRX has previously been demonstrated in Schwann cells of mouse peripheral nerves but not the central nervous system. We used the same antisera against PRX (which highlighted human endothelium) to stain brain from several non-human species: two strains of mouse, rat, pig, and marmoset. No brain PRX expression was detected in those species (Fig. 1E shows negative mouse brain staining). In mouse kidney, vascular structures, including the glomeruli, did not stain (Fig. 1F), but perivascular nerve bundles showed reactivity (Fig. 1G). The PRX antibody also strongly recognized lung capillaries (Fig. 1H). In the mouse tail, nerve bundles and endothelial cells were stained with the PRX antibody (Fig. 1I). These studies demonstrated that the polyclonal antibodies to PRX recognized both human and mouse protein but that there is human-specific expression in the brain endothelium.

**Figure 2:**
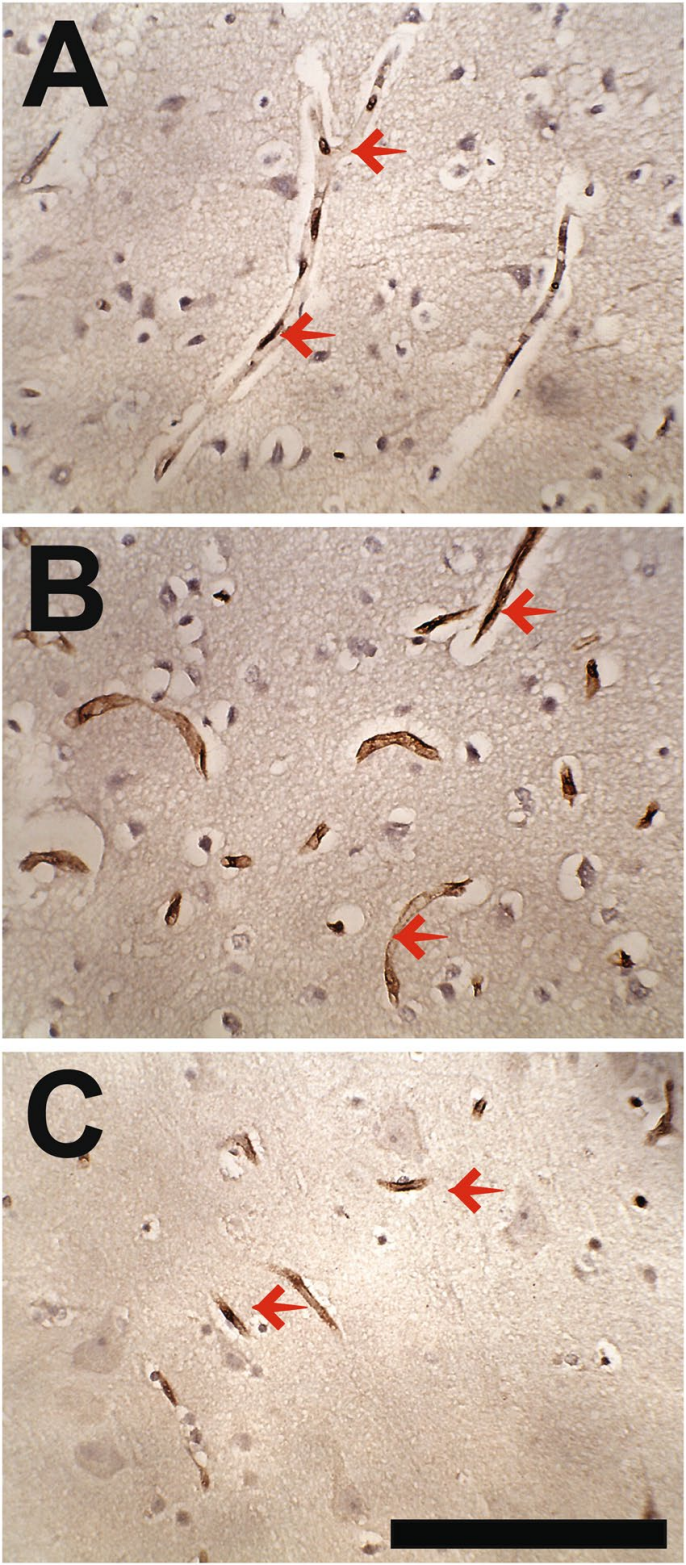
Expression of PRX in capillaries in young human brain. Immunohistochemical analysis of human brain confirmed the expression of PRX over a wide span of ages. Capillary staining (left pointing arrows) similar to that seen in aging human brain was observed in brains from individuals at 3 months (**A**) 6 years (**B**) and 18 years (**C**) of age. Capillary cells in (**A**) show an abundance of staining in a nuclear pattern. Scale bar shows 150 microns for all photographs.

To determine whether PRX is present in young humans, we stained a different cohort of human brains ranging in age from 2 months to 18 years. We found expression in cortical capillaries in all human samples, regardless of age (Fig. 2). Notably, in many but not all vessels, PRX reactivity localized heavily to endothelial cell nuclei.

**Figure 3:**
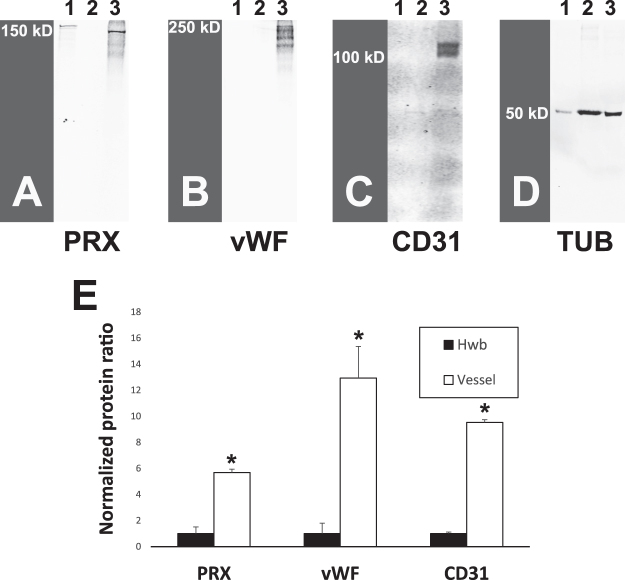
Expression of PRX protein in human brain vessel fractions. Fractions of human brain from autopsy were analyzed for presence of indicated proteins. Lane 1: 293 cells transfected with L-PRX full length cDNA (encoding Genbank Protein ID AAH67266.1; purchased from OriGene); Lane 2: Human whole brain (Hwb) lysate; Lane 3: Partially purified microvessel proteins from human brain. Lane 1 was underloaded to avoid overloading PRX. (**A**) Western blotting of brain vessels showed expression of a protein band corresponding to the larger form of PRX (L-PRX), as seen in transfected 293 cells. As controls, the same protein lysates were probed for (**B**) vWF, (**C**) CD31, and (**D**) tubulin. Quantification of the degree of enrichment of vascular proteins in vascular fractions was computed by first calculating the ratio of the protein of interest to tubulin (**D**) and then normalizing this ratio in vessels to the ratio in human whole brain. (**E**) shows overall levels of protein enrichment in vessel fractions of multiple individuals (n = 3 for PRX and vWF and n = 2 for CD31; *indicates p < 0.01). Additional brain samples and higher exposure PRX blots are shown in Supplemental Fig. 1.

We also assessed the expression of PRX protein in vascular tissue by Western blotting. Crude vessel fractions were prepared from human cortical tissue. Vascular fractions showed substantially increased expression of a major band corresponding to the predicted molecular mass of full length L-PRX, the larger of two known gene products (Fig. 3). On longer exposure of the blot, a cluster of protein sizes were observed in cells transfected with L-PRX cDNA, whole brain, and brain vessel preparations. The major band from transfected cells was slightly higher than the brain bands, but all samples demonstrated clusters of high molecular weight sizes that demonstrated overlap as shown in Supplemental Fig. 1. The degree of PRX enrichment in vascular fractions of the brain was similar to that of established endothelial markers, vWF and CD31 (Fig. 3E).

### Subcellular expression of PRX in brain endothelial cells

**Figure 4:**
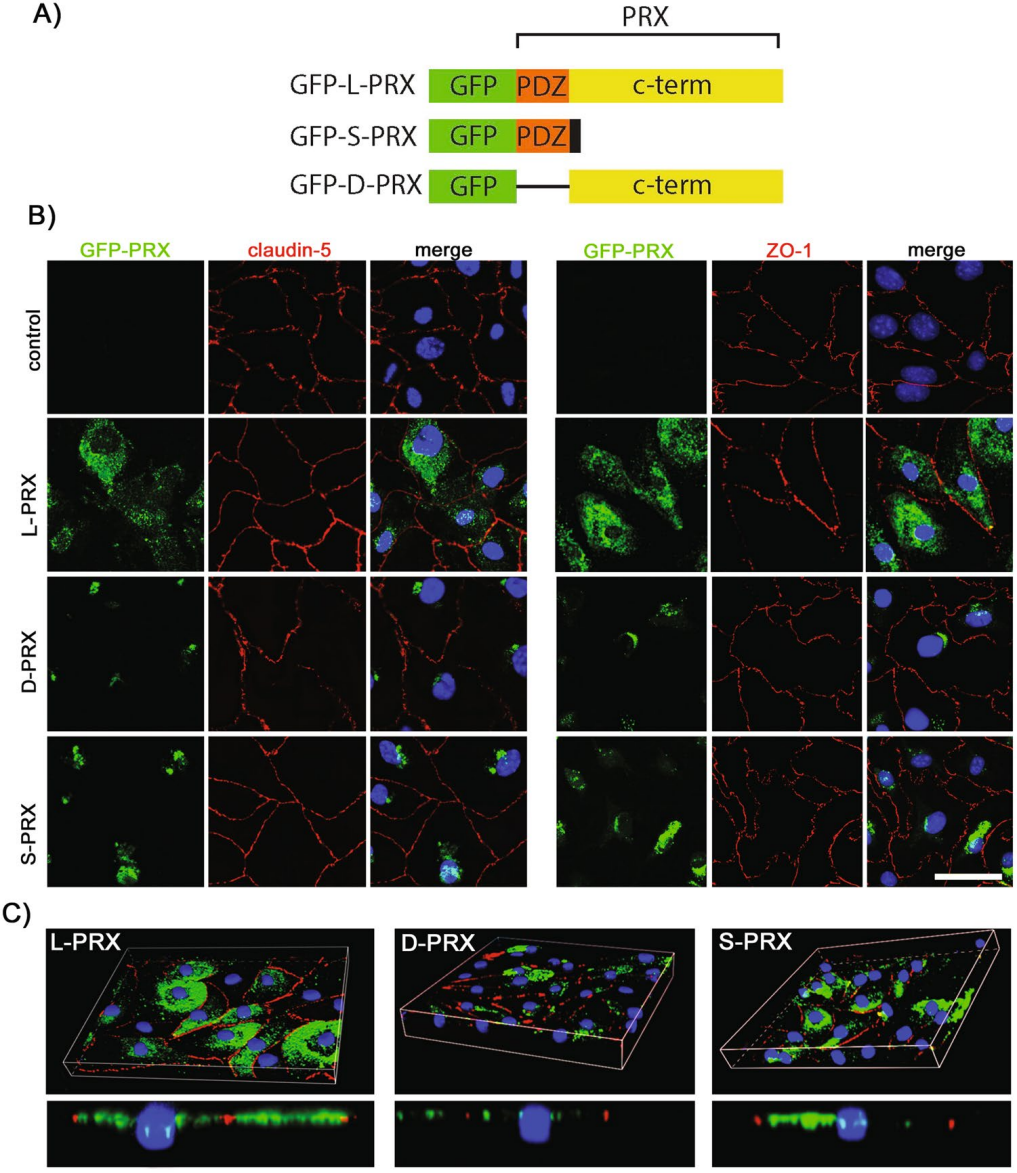
Subcellular localization of PRX in cerebral endothelial cells. Primary mouse brain endothelial cells were transfected with GFP constructs (**A**). L-PRX is the full length form of PRX which includes a PDZ domain and a long, repetitive C-terminal sequence. S-PRX is short form that is composed principally of the PDZ domain and is generated naturally by alternative splicing. The D-PRX construct, which deletes the PDZ domain from L-PRX, was used to determine cell localization mediated by sequences outside of the PDZ domain. Cells were fixed and then stained for claudin-5 and ZO-1, with DAPI to highlight nuclei (**B**). (**C**) Z-stack images were processed in order to assess localization of the three PRX constructs in three dimensional space. Scale bar measures 50 microns.

Because immunohistochemical studies suggested PRX could be localized to both cell nuclei and cytoplasm in human brain, we examined the subcellular expression of PRX protein in finer detail in cultured endothelial cells. The large form of PRX is composed of an N-terminal PDZ domain, a middle repeat domain, and a C-terminal domain. To map regions of PRX that could contribute to subcellular localization, we prepared GFP fusions (Fig. 4A) to PRX corresponding to the full length PRX (L-PRX), a splice form of PRX which generates a short form (S-PRX) that contains the PDZ domain of the protein and very small C-terminal tail^21^, and a construct that deletes the PDZ domain from the full length protein (D-PRX, which does not exist naturally). Constructs were expressed in primary mouse cerebral endothelial cells, and GFP was examined along with the tight junction markers claudin-5 and ZO1 (Fig. 4B). GFP-L-PRX was found largely in the nuclei of mouse endothelial cells. A modest amount of L-PRX was found in the cytoplasm and very little L-PRX was located at the cell membrane where the predominance of ZO-1 protein is enriched. The small isoform of PRX (S-PRX), expressed as a fusion protein to GFP was localized outside of the nucleus of endothelial cells, with a minor component of protein seen in the nucleus. There was very little GFP-S-PRX at the plasma membrane of transfected cells. To test for nuclear localization signals in the C-terminus of PRX, we also tested the localization of a GFP fusion to PRX sequences that are not present in S-PRX. We found that this protein (GFP-D-PRX; Fig. 4B) had a similar distribution to S-PRX. Confocal Z-stack images confirm that endothelial cells expressing PRX harbor proteins that reside within the nucleus rather than on the surface (Fig. 4C). We concluded that L-PRX is mostly found in the cytosol and nucleus and that, in endothelial cells, nuclear localization cannot be accounted for by a single dominant nuclear localization sequence. Transfection of untagged PRX construct into mouse cerebral endothelial cells resulted in similar, robust localization to the nucleus in a population of cells (Supplemental Fig. 2), and HEK293 expression of recombinant PRX resulted in protein expression in nuclear fractions of cellular protein preparations (Supplemental Fig. 3).

### Gene expression alterations triggered by endothelial PRX

**Figure 5:**
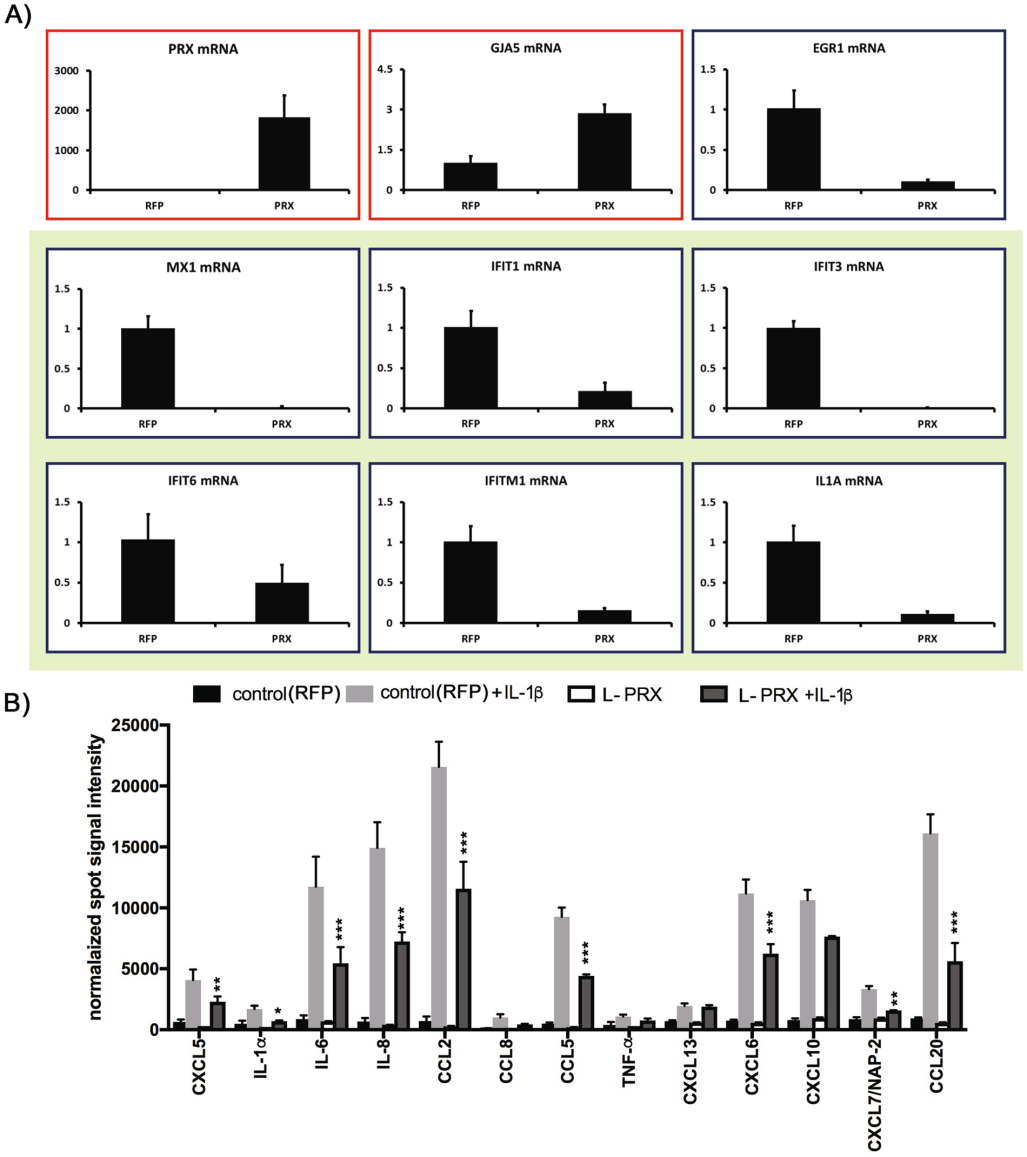
Effect of PRX expression on inflammatory markers in endothelial cells. (**A**) Effect of PRX on inflammatory factor mRNA levels in endothelial cells. Human primary cerebral endothelial cells were infected with L-PRX viruses or RFP control viruses. After 24 hours, RNA was analyzed by quantitative RT-PCR by normalizing Ct values to 18S rRNA levels. Expression of a series of inflammatory mRNA species (green background) was significantly suppressed, as shown. Few mRNA species (red background) were upregulated. Samples were analyzed in triplicate with error bars shown as SD; all comparisons between PRX and RFP infected samples were significant with p < 0.05 (t-test). Experiments were repeated showing the same differences with three sets of cell cultures. (**B**) Effect of PRX on human cerebral endothelial protein expression after inflammatory challenge. We display protein array signals for human cytokines present in control (RFP-transfected cells) and L-PRX overexpressed HBMEC treated with/without IL-1β (10 ng/ml). Data represent several highly increased cytokines/chemokines in cell culture media collected at 24 hrs after introducing IL-1β. L-PRX presence in brain endothelial cells decreased expression of several proinflammatory cytokines. Values are means ± SD, n = 3; *p > 0.05, **p > 0.01, ***p > 0.001.

Global gene expression analysis was performed to determine the impact of PRX expression in human cerebral endothelial cultures. Microarray analysis of RNA from PRX-expressing cells (relative to control cells expressing RFP) revealed significant increases in a small fraction of transcripts, shown in Table 1 (top). On the other hand, a larger number of transcripts were significantly decreased by expression of L-PRX (Table 1, bottom). A large fraction of transcripts decreased after L-PRX expression participates in inflammatory responses, and, in particular, the five most strongly suppressed genes are canonical members of the Type I interferon response pathway (Table 1). Validation experiments using independent sets of cultured human endothelial cells expressing L-PRX or RFP demonstrated strong suppression of IFIT1, IFIT3, IFIT6, IFITM1, IL1A, and MX1(Fig. 5A). TNF-alpha and IL1-beta transcript levels were not affected (Supplemental Fig. 4). On the other hand, in the same transcriptome experiment, no endothelial tight junction factors were increased by more than 1.5 fold after expression of L-PRX (Table 2).

**Table 1:** Genes in human brain endothelial cells regulated by PRX expression.

Probe Set	Gene	Activation (PRX/control)	Repression (control/PRX)	Role in inflammation	IFN response pathway
***Upregulated***					
16872430	PRX	40.88	0.02		
16870978	ZNF675	3.84	0.25		
17104049	SNORA11	3.71	0.26		
17068905	PCMTD1	2.54	0.39		
16826892	CSNK2A2	2.35	0.42		
***Downregulated***					
17005515	TRIM38	0.48	2.05		Type 1
17118413	FAM27E3	0.42	2.33		
16850428	ROCK1P1	0.42	2.36		
16925023	SNORA80A	0.42	2.37		
16852683	PMAIP1	0.38	2.61		
16790229	LINC00641	0.38	2.62		
16797419	IGHG1	0.34	2.88	Yes	
16684080	IFI6	0.34	2.91	Yes	Type 1
16901974	IL1A	0.34	2.93	Yes	
16989736	EGR1	0.29	3.41	Yes	
17004612	DSP	0.27	3.58		
16904365	IFIH1	0.27	3.68	Yes	Type 1
16666485	IFI44L	0.26	3.72	Yes	Type 1
16923031	MX1	0.26	3.8	Yes	Type 1
16707184	IFIT3	0.22	4.49	Yes	Type 1
16707196	IFIT1	0.13	7.42	Yes	Type 1

Microarray analysis was performed on RNA from two paired sets of endothelial cells that were infected with either PRX or RFP encoding lentiviruses. Twenty one genes were identified that were regulated by two-fold or more between both pairs of samples. For ease of interpretation, we calculated the relative degree of induction (PRX/RFP values) and the repression (RFP/PRX values). A role in inflammation or interferon response pathway, as annotated in uniprot.org, is also shown.

**Table 2:** Regulation by PRX of genes encoding blood brain barrier proteins.

Probe set	Gene	Descriptive Name	Average PRX/control	Protein type	Number of PDZs
16932321	CLDN5	claudin-5	1.48	TM	
16712854	MPP7	Membrane Palmitoylated Protein 7	1.38	Adaptor	1
16801400	CGNL1	Cingulin Like 1	1.31	Adaptor	
16695490	F11R	Junctional adhesion molecule A	1.3	TM	
16668919	MAGI3	Membrane-Associated Guanylate Kinase	1.28	Adaptor	6
16913315	DLGAP4	DLG Associated Protein 4	1.25	Adaptor	
16985704	OCLN	occludin	1.24	TM	
16819794	CDH5	Ve-cadherin	1.24	TM	
16745693	ESAM	Endothelial Cell Adhesion Molecule	1.23	TM	1
16985688	MARVELD2	MARVEL Domain Containing 2	1.2	TM	
17082263	SCRIB	Scribbled Planar Cell Polarity Protein	1.19	Adaptor	
16746591	WNK1	Serine/threonine-protein kinase WNK1	1.18	Adaptor	
17009008	TJP1	Zonula occludens protein 1 (ZO-1)	1.13	Adaptor	
17115692	MPP1	Membrane Palmitoylated Protein 1	1.1	LA	1
16889845	PARD3B	Par-3 Family Cell Polarity Regulator Beta	1.07	Adaptor	3
17048188	CLDN12	claudin-12	1.05	TM	
16856159	PARD6G	Par-6 Family Cell Polarity Regulator Gamma	1.04	Adaptor	1
16806467	TJP1	Zonula occludens protein 1 (ZO-1)	1.04	Adaptor	3
16730268	AMOTL1	Angiomotin Like 1	1.04	Adaptor	
16963455	DLG1	Discs Large MAGUK Scaffold Protein 1	1	Adaptor	
17085685	TJP2	Zonula occludens protein 2 (ZO-2)	0.99	Adaptor	3
16825154	ARHGAP17	Rho GTPase Activating Protein 17	0.97	Adaptor	
16921832	JAM2	Vascular Endothelial Junction-Associated Molecule	0.97	TM	
17048167	CLDN12	claudin-12	0.97	TM	
16785606	MPP5	Membrane Palmitoylated Protein 5	0.97	Adaptor	1
16712825	MPP7	Membrane Palmitoylated Protein 7	0.95	Adaptor	1
17113418	AMOT	Angiomotin	0.95	Adaptor	
16736847	LIN7C	Protein lin-7 homolog C	0.94	Adaptor	1
17048165	CLDN12	claudin-12	0.92	TM	
16959441	AMOTL2	Angiomotin Like 2	0.85	Adaptor	
16713230	PARD3	Partitioning defective 3 homolog	0.76	Adaptor	3

Level of PRX regulation is shown for elected genes that encode proteins implicated by Daneman *et al*. (Daneman *et al*. 2010) to participate in BBB function. We extracted averaged values of PRX/RFP expression levels from the same dataset from human cerebral endothelial cells used to generate Table 1. Protein type and the presence of PDZ domains in each protein was extracted from uniprot.org. CLDN12 and MPP7 are listed more than once since they have more than one probe set on the microarray. JAM4 and ASHL1 were not represented. TM = Transmembrane. LA = Lipid Anchored.

### Secreted cytokine/chemokine expression in endothelial cells after inflammatory challenge

The potential effect of PRX on inflammatory cytokines was assessed in human cerebral endothelial cells that were challenged with IL-1b. Cells were infected with viruses expressing PRX or RFP (as a control) and after 48 hours, were exposed to IL-1b. The secreted proteins from the media were analyzed using a protein based antibody array for human cytokines. Selected cytokines were activated by IL-1b but suppressed by the expression of PRX. In total, of the 80 cytokines analyzed, 22 predominantly proinflammatory cytokines/chemokines were suppressed by PRX expression. As such, the inflammatory suppressive effect of PRX extends beyond suppression of gene expression and results in decreased protein release (Fig. 5B).

### Endothelial PRX strengthens BBB function

**Figure 6:**
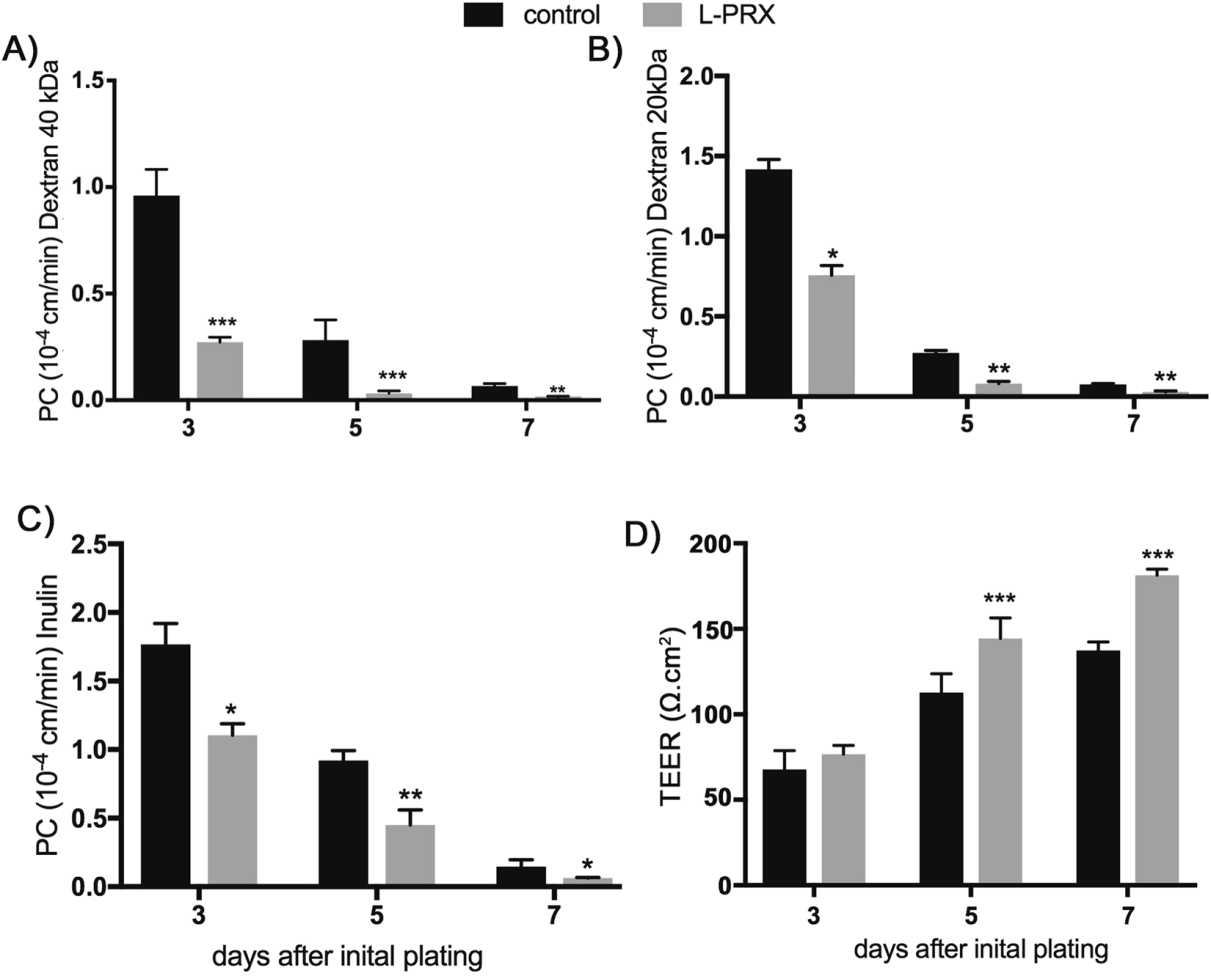
Effect of PRX on mouse cerebral endothelial cell permeability. L-PRX was expressed in primary mouse cerebral endothelial cells using recombinant lentiviruses. Uninfected cells were used as a control. At the number of days after infection indicated on the x-axis, permeability to indicated fluorescent tracers was determined (**A**–**C**). (**D**) TEER was also determined at the same time points. L-PRX significantly strengthened the barrier function of endothelial cells, accelerating both the acquisition of barrier function and the final resistance of cells to tracers of a wide range of molecules. RFP lentivirus infected cells showed the same permeability profiles as uninfected cells (not shown). Data represent means +/− SD for n = 5 independent experiments. *p < 0.05, **p < 0.01, ***p < 0.001 comparing control and L-PRX transfected cells.

To test if PRX has a role in barrier function, we investigated potential effects of PRX on primary mouse cerebral endothelial cell barrier function. Cells expressing L-PRX (versus control cells shown) for up to 7 days were grown in transwell chambers. Cell barrier function was assessed in permeability assays using three different size tracers (Inulin 5 kDa, Dextran 20 kDa and Dextran 40 kDa). As shown in Fig. 6, beginning at day 3 after introduction of the transgene, the expression of L-PRX significantly reduced permeability to all three tracers, significantly enhancing monolayer barrier function at days 5 and 7. This was most significant for, Dextran 40 kDa (p < 0.001) and Dextran 20 kDa (P < 0.01). In addition, transendothelial electrical resistance (TEER) progressively increased during each day of observation with significant increases in tightness at days 5 and 7 (p < 0.05). RFP lentivirus infected cells showed the same permeability profiles as uninfected cells. In sum, expression of the human protein L-PRX in mouse cerebral endothelial cells exhibits significant barrier strengthening properties.

### PDZ domain is necessary and sufficient for barrier function modification

**Figure 7:**
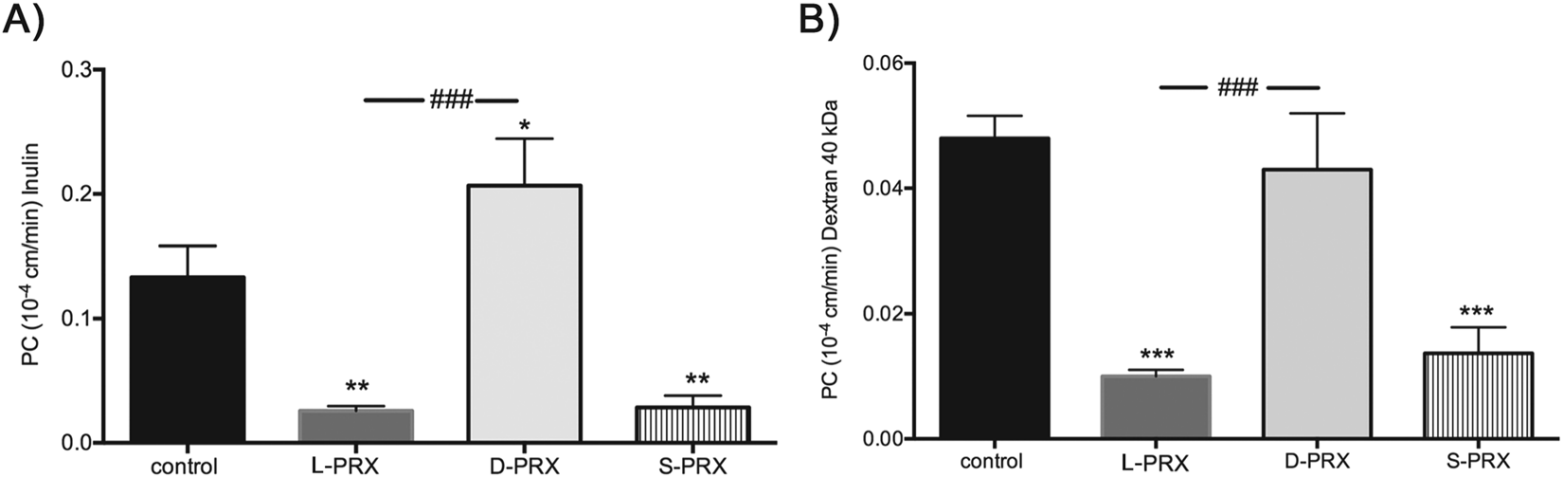
Mapping structural domains of PRX that mediate enhancement of endothelial permeability. The effect of three components of PRX on endothelial permeability was compared. Lentiviruses were used to express either full length L-PRX, S-PRX, or D-PRX (C-terminal PRX) (similar to the sequences shown in Fig. 4A, minus the GFP tag) and permeability was measured as in Fig. 6 for each group of infected primary mouse brain endothelial cells. Permeability to both inulin (**A**) and dextran (**B**) were measured. Enhancement of barrier function was observed in cells expressing S-PRX and full length L-PRX, but not with D-PRX. Uninfected cells served as controls. RFP lentivirus infected cells showed the same permeability profiles as uninfected cells (not shown). Data represent means +/− SD for n = 3 independent experiments. *p < 0.05, **p < 0.01, ***p < 0.001, comparing control and S-, D- and L-PRX transfected cells. ^###^p < 0.001 comparing L-PRX with S- and D-PRX transfected cells.

To map domains of the PRX coding sequence which mediate barrier strengthening function, we generated recombinant viruses encoding S-PRX and D-PRX (and N-terminal truncation that eliminates the PDZ domain). We compared mouse cerebral endothelial cells infected with these viruses to cells infected with full length L-PRX for permeability (Fig. 7). Cells expressing S-PRX had improved barrier function to all tracers and increased TEER compared to control cells. The enhancement of function was similar that induced by expression of full length (L-PRX). In contrast, D-PRX did not have an impact on endothelial permeability.

### PRX blunts inflammation driven endothelial permeability

**Figure 8:**
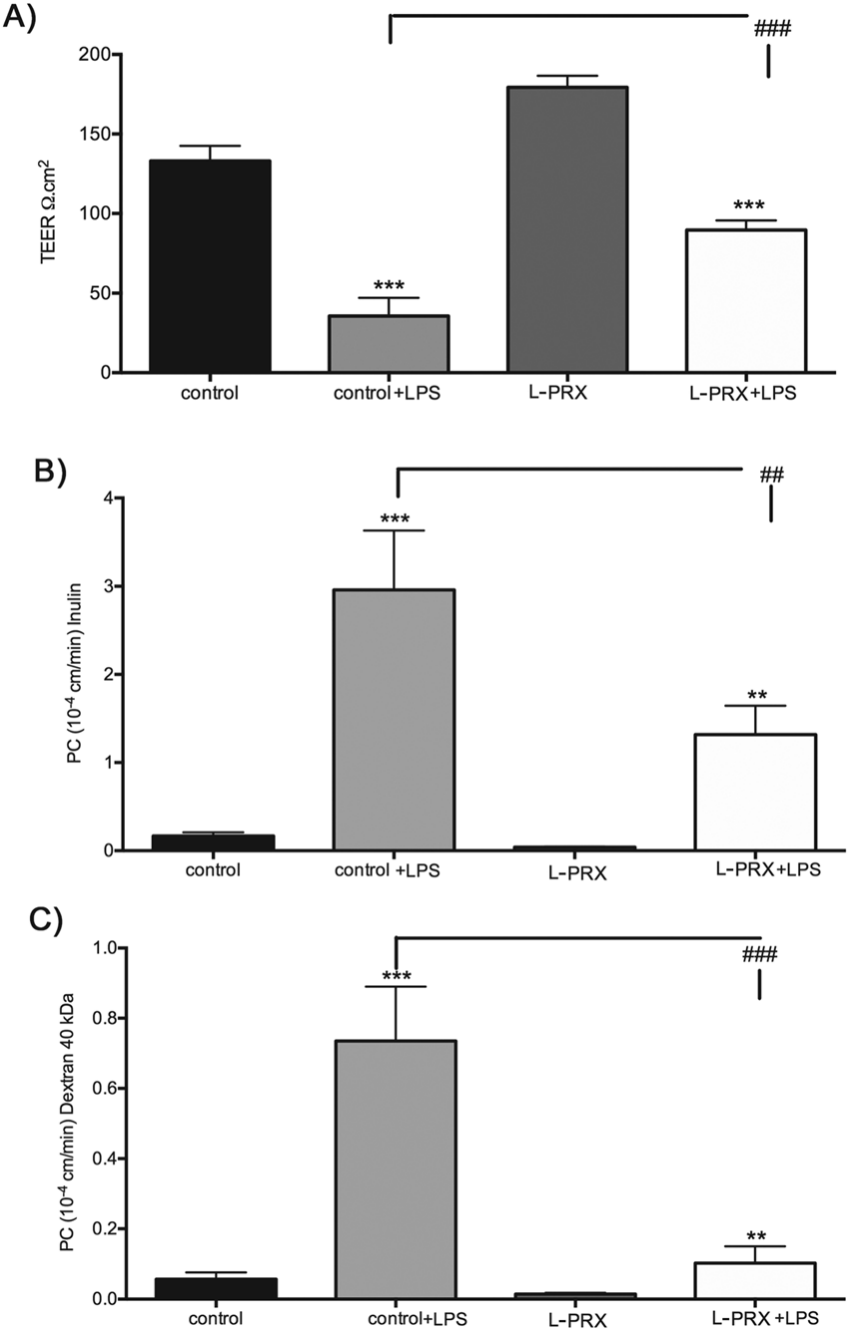
PRX as an attenuator of inflammatory elevation of endothelial permeability. Murine endothelial cells were infected with viruses to express L-PRX. Afterwards, cells were challenged with LPS (5 ug/ml), which stimulates endothelial permeability. Measurements of permeability to a series of tracer proteins shown and of TEER demonstrate that L-PRX reduces LPS-stimulated permeability to proteins of a range of molecular weights. Uninfected cells served as controls. RFP lentivirus infected cells showed the same permeability profiles as uninfected cells (not shown). Data represent means +/− SD for n = 3 independent experiments. **p < 0.01, ***p < 0.001, comparing LPS-challenged to control cells. ^###^p < 0.001 comparing L-PRX expressing to control cells.

Inflammatory mediators such as cytokines and bacterial products (e.g. lipopolysaccharide; LPS) cause increased endothelial permeability. Since PRX mediates coordinated repression of inflammatory response genes, we determined whether PRX expression could counteract inflammatory factor-mediated increased endothelial permeability. Control endothelial cells exposed to LPS demonstrated 3–7 fold increases in permeability to three tracers in transwell assays. RFP lentivirus infected cells showed the same permeability profiles as uninfected cells (not shown). L-PRX expression resulted in a marked suppression of inflammatory factor-stimulated permeability (Fig. 8). Cells expressing L-PRX resisted challenges to barrier function, although this function was not fully normalized. As before, S-PRX also led to resistance to inflammatory challenge, but D-PRX (lacking the PDZ domain) did not exhibit barrier enhancing function.

## Discussion

A complex array of molecular components regulates cerebral endothelial properties. This study identified PRX as a new protein expressed in human cerebral endothelial cells that is capable of regulating cell barrier physiology. The core findings of our studies are that: (1) PRX is unique among vascular PDZ-domain containing proteins in that it is present in human (but not other vertebrate) brain endothelial cells and that it does not preferentially localize to tight junctions; (2) PRX sharply down-regulates gene and protein expression related to inflammation; and (3) PRX markedly enhances barrier properties of endothelial cells and suppresses inflammatory-stimulated increases in endothelial permeability.

### Motivation for human cerebrovascular protein identification

Use of non-human species to model cerebrovascular disease such as stroke has largely fallen short in yielding information that has advanced therapy. The reasons for the disconnection between animal and human clinical trials have been debated with vigor, and an often overlooked consideration is that vascular protein content may have species differences. PRX was among the first of over a dozen factors discovered to be human-only cerebrovascular markers in an ongoing database screen for novel human cerebrovascular factors. This effort, which is still ongoing, involves the manual annotation of human cerebral protein expression patterns^20^, publically available on the Human Protein Atlas^22^. The identification of PRX as a *functional* BBB protein that is present in humans (but not in traditional experimental animals) suggests that vascular protein expression differences could account for limitations of animal models.

A small number of proteins have already been demonstrated as human-enriched or human-specific in endothelial cells^23,24^ and Schwann cells^25^. Our new characterization of PRX demonstrates, for the first time to our knowledge, that a human-specific factor modifies cerebral endothelial cell permeability. Future studies, such as the generation of mice expressing human PRX and other human-only factors, will allow critical testing of the possibility that humanizing the cerebrovascular will advance the predictive value of mouse models of stroke and other brain blood vessel disorders.

### PRX expression in the nervous system

Previous work in mouse has demonstrated PRX in Schwann cells and the lens^26^ but not the central nervous system^27^. The function of PRX has been elucidated in mouse and human genetic studies which show that it plays an essential role in the establishment of functional myelin and lens fiber structure^26,28^. Rare cases of human PRX loss of function mutations cause recessive peripheral neuropathies, including Dejerine-Sottas syndrome^29^ and Charcot-Marie-Tooth disease^30,31^. These studies firmly establish PRX as an essential protein in the peripheral nervous system across species.

In the Human Protein Atlas, PRX antibodies stain peripheral nerves of humans; furthermore, staining of multiple organs of the mouse, in this study, confirm that PRX is expressed robustly in the peripheral nervous system. The strong localization of PRX to the same structures shows that the antibodies used in this study recognize the protein in multiple species.

We used these antibodies to demonstrate that PRX is expressed in human capillaries of the brain and that it is not expressed at detectable levels in brain of a variety of other species. Further confirmation of PRX expression in human brain vessels is provided by in situ hybridization and by Western blotting of vessel-enriched protein preparations. While the human expression of PRX in the brain has not previously been reported, our inability to find PRX in mouse brain confirms previous studies^27^, which employed mRNA detection in tissues. We emphasize that in addition to its absence in the mouse, PRX was not found in the brain vasculature in a variety of species, including the non-human primate, marmoset, that is sometimes used as an experimental model. Because antibodies recognize both mouse and human PRX proteins, we believe that the antibodies should recognize PRX in most other species that are more closely related to human because of substantial homology (similarity of human vs. mouse [84.1%], vs. rat [82.9%], vs. pig [86.4%], vs. marmoset [95.2%]). A potential limitation of our study is that antibodies were generated to the C-terminus of PRX that is present only in L-PRX. Thus, we cannot address the species distribution of the minor S-PRX form, though we can ascertain that L-PRX is human specific for the brain endothelium.

The subcellular distribution of PRX within cerebral endothelial cells distinguishes it from other PDZ-domain containing proteins that regulate junction function. Those proteins localize to the cell-cell junctions and are thought to play key roles in formation of the BBB by physically stabilizing these structures. In myelinated peripheral nerve, PRX is localized to appositions between myelin and the plasma membrane that define Cajal bands^32^. Thus, we expected to find at least a fraction of PRX associated with the plasma membrane and with the tight junction proteins claudin-5 and ZO-1. In contrast, we found no junctional PRX and a large fraction of nuclear PRX in endothelial cells, raising the possibility of a role for PRX in transcriptional processes (see below).

### Potential molecular mechanisms of action of PRX

PRX effected substantial enhancement of cell barrier function, a key property of brain endothelial cells. The increase in barrier strength was consistent across a wide range of molecules of diverse molecular weights, including ions (measured by TEER). PRX is an unusual example of a single regulatory protein whose overexpression strengthens baseline endothelial barrier function and inhibits the deleterious effects of an inflammatory stimulator (i.e. LPS) on endothelial barrier function.

Specific immediate signaling pathways by which PRX acts in endothelial cell are suggested by comparisons between S-PRX, L-PRX, and D-PRX. Since the PDZ domain of PRX is both necessary and sufficient for its barrier enhancing function, proteins that bind to the PDZ domain of PRX likely initiate molecular events that improve endothelial barrier function.

Proteins sequences that bind to PDZ domains are generally located at the C-termini of target proteins. Other PDZ proteins in endothelial cells generally target tight junction proteins and are thought to directly participate in structures that strengthen barrier function. But, we identified PRX principally in the nucleus and cytoplasm and failed to find localization with either claudin-5 or ZO-1. Prior work showed expression of PRX in the nucleus and that control of subcellular localization was responsive to a nuclear export signal within S-PRX^33^ and by nuclear localization signals in the PDZ domain^34^. Our results suggest that in agreement with previous studies, nuclear localization signals within the PDZ domain influences cell distribution of PRX in cerebral endothelium.

In the absence of colocalization with claudin-5 or ZO-1, available evidence suggests an alternative role for the PDZ domain of PRX which may affect nuclear function in addition to (or instead of) tight junction complexes. Gene expression studies of endothelial cells demonstrate that intermediate events stimulated by PRX predominantly reflect suppression of genes; genes suppressed more than 2-fold vastly exceeded those activated more than 2-fold. Furthermore, the most strongly suppressed genes were factors that have been implicated in response to inflammation and infection. Inflammation and infection, in turn, are generally associated with increases in cerebral vascular permeability. In accordance, inflammatory stimulation of levels of a wide array of proteins was attenuated by PRX expression in endothelial cells. The link between PRX-induced gene suppression and PRX-mediated permeability suppression may thus conceivably be functionally linked. Our findings are also congruent with the known effects of corticosteroids, first line therapy for reducing BBB permeability caused by vasogenic brain edema^35,36^ and potent global inhibitors of inflammatory gene expression.

Surprisingly, we did not find evidence for notable transcriptional upregulation of tight junction genes in PRX expression studies (Table 2). Among proteins thought to play a role in endothelial permeability, we did not find more than 1.5-fold increases in expression of any gene encoding tight junction proteins, and a similar number of tight junction genes were up- and down-regulated. In aggregate, these studies imply that PRX potentially mediates tight junction tightening via post-transcriptional effects, which may include protein phosphorylation^37^ and other post-translational controls of tight junction proteins^4^.

### PRX in normal physiology and disease

Brain viability clearly does not require endothelial expression of PRX, since all non-human species examined do not express this protein. In addition, loss of function PRX mutations in humans cause a demyelinating peripheral neuropathy, but there are no symptoms that can be attributed to impaired cerebral endothelial function reported in affected individuals. At first approximation, this may suggest that the impact of PRX on the brain endothelium is minimal. However, it is known that some components of endothelial tight junctions are frequently not required for *in vivo* function of the blood brain barrier (for example ZO-3^38^). It remains possible that PRX plays a homeostatic role under perturbed states (e.g. during inflammatory challenges and ischemia).

An important impetus for studying proteins that modify the BBB is to identify potential control points that can be leveraged to modulate cerebral endothelial function in disease. In brain conditions that require medications to cross the BBB, impairing barrier function could be beneficial. In other conditions, such as brain injury due to trauma, stroke or dementia, tightening a dysfunctional BBB is advantageous. To control BBB function, identification of a protein that is not absolutely required for BBB function but whose levels can increase or decrease BBB function would be attractive. As a protein that is clearly not essential for BBB function in other species, but that increase barrier function of endothelial cells, PRX may be positioned favorably as a target for development of approaches to control the human BBB during pathological states. From a practical perspective, PRX, if delivered in an active state, could offer substantial advantages over current medications for treating increased brain vascular permeability. Corticosteroids, while effective, affect many other cells and thus induce significant side effects. PRX and pathways stimulated by this protein may offer alternative targets for BBB modification that act on the same pathways as endothelial cell glucocorticoid receptors.

## Methods

### Animal studies

This study used vertebrate animals. All experiment that involved animals were performed in accordance with NIH guidelines using protocols approved by the Institutional Animal Use and Care Committee (IACUC) at the University of Michigan and the VA Ann Arbor Healthcare System.

### Immunohistochemistry

Formalin fixed frontal lobes sections were obtained from the Alzheimer’s Disease Center at the University of Michigan and the Brain Bank of the National Institute for Developmental and Childhood Disorders at the University of Maryland^39^. Five micron sections were analyzed using chromagenic immunohistochemical staining using a rabbit polyclonal antibody to PRX (Sigma, St. Louis, MO) after citrate-induced antigen retrieval. Staining was followed by hematoxylin counterstaining. Human tissue antigen integrity was ascertained by staining with the mouse monoclonal antibody BRIC231 (anti-H; Santa Cruz). Integrity of mouse tissue antigens was evident in most tissue blocks because PRX is uniformly expressed in peripheral nerve bundles in all organs.

### *In situ* hybridization

Localization of PRX in human brain was performed using a chromogenic detection method (Advanced Cell Diagnostics)^40^. Hybridization of antisense nucleic acid probes against PRX was followed by multiple non-enzymatic amplification steps, and, finally, probe detection was accomplished with an alkaline phosphatase conjugated terminal probe. After colorimetric detection of the *in situ* signal, we counterstained with hematoxylin. Brain blocks that resulted in signal with ubiquitous positive control probes were selected for this study.

### DNA constructs and virus production

Full length, human PRX cDNA was generated by PCR from a commercially obtained clone (Origene) and cloned into the NotI and XbaI sites of the pLENTI-EV vector for expression studies; the long form in this study is designated L-PRX. D-PRX was generated by PCR using primers to delete sequence encoding the first 199 amino acids of the N-terminus of PRX, replacing this with a Kozak sequence to initiate translation. S-PRX was generating using PCR from the original template and inserted into the NheI and BamHI sites of pLENTI-EV; a unique sequence encoding the short C-terminal tail of S-PRX and stop codon were engineering into the PCR primer. Corresponding GFP fusion clones were generated by inserting these PRX fragments into pEGFP-C3 (Clontech), resulting in a fusion of EGFP at the N-terminus of the PRX sequences. These clones were inserted into XhoI (Klenow blunted) and EcoRI sites of the vector and NcoI (Klenow blunted) and EcoRI sites in the inserts. As a control for viral infection, lentiviruses encoding RFP were also generated.

### Western blotting

Whole brain and vessel-enriched fractions were prepared as follows^41^: All procedures were performed on ice or at 4 °C. Frozen human brain tissue from autopsies (10 g) was disrupted in 15 ml B1 buffer (HBSS with HEPES (10 mol/L)) using a type A glass dounce homogenizer. The brain lysates were centrifuged at 2000g for 10 min, and the cloudy supernatant decanted. The pellet was resuspended in 20 mL of B1 with 18% dextran, and centrifuged at 4400 g for 15 min. The myelin-rich layer and dextran supernatant were removed, and the microvessel pellet was resuspended in 6 ml B3 buffer (B1 supplemented with 1% bovine serum albumin). The microvessels were then passed through 70 μM nylon mesh cell strainers (BD Falcon). Microvessels in the filtrate were washed with B3 and collected in 20 ml B3, centrifuged at 2000g for 5 min, and the pellet was resuspended in 1 mL of B3 and transferred into a microcentrifuge tube, pelleted, and resuspended in RIPA buffer.

Standard electroblotting was performed as before^42^. Fractions were electrophoretically separated on SDS polyacrylamide gels, electrotransferred to nitrocellulose, and probed using PRX or vascular control antibodies (Sigma, St. Louis, MO) at 1:1000 dilution. Labeled secondary antibodies were detected using a Licor Odyssey infrared scanner. Expression levels were normalized to tubulin content assessed on either the same filter or a parallel Western blot.

### RNA quantification

Quantitative reverse transcriptase PCR was performed and analyzed as before^43^. Cells were scraped off plates and then lysed in lysis buffer per manufacturer’s protocol. RNA was purified using an RNeasy spin column kit (QIAGEN). We converted RNA by reverse transcription and then cDNA was quantified by real time PCR, using HPRT, as a control to assess target gene regulation.

### Human brain microvascular endothelial cell culture

Primary Human Brain Microvascular Endothelial Cells (ACBRI 376) were purchased from Cell Systems Corporation (Kirkland, WA 98034, U.S.A). Cells were cultured in CSC Complete Medium which includes 10% fetal bovine serum. The day before recombinant lentiviral infection, cells were split to 48-well-plates coated with CSC Attachment Factor. On the day of infection, 50 μl lentivirus stock was diluted into 0.5 ml CSC complete media; 1 μl of 4 mg/mL polybrene was added to the diluted virus to a final concentration of 8 μg/mL; this mixture was then used to replace the cell media. The viral media was replaced with CSC Complete Medium after 1 day of infection. After an additional day of culture, cells were harvested for RT-PCR. As a control for gene expression studies, we analyzed human brain endothelial cells that were infected with lentivirus encoding RFP.

### Microarray analysis

Transcriptome analysis was performed using Affymetrix Human Gene ST 2.1 strips that were processed in the University of Michigan Microarray Facility. Labeled cDNA was prepared using a WT Pico Kit (Affymetrix). Probesets that yielded differences of more that 2-fold between PRX and control groups were considered potentially differentially expressed. Two independent replicates were performed, and the 66 genes that overlapped between two experiments were presented in the final results. A subset of these genes were assessed by quantitative RT-PCR to validate in independent cell groups, performed in triplicate, whether expression was altered by PRX expression.

### Protein array analysis

A human cytokine antibody array kit (RayBiotech Inc) was used to simultaneously detect and quantify 80 cytokines/chemokines in cell culture media samples. Briefly, human brain endothelial cells with or without overexpressed PRX, were exposed to Il-1b (10 ng/ml, Preprotech) for 24 hrs, and cell culture media was sampled. The antibody array was performed according to the manufacturer’s instructions. Densitometric analysis was performed using ImageJ analysis software using a protein array analyzer plugin. The relative level of cytokines was evaluated using software provided by the manufacturer.

### Mouse brain microvascular endothelial cell culture

Mouse brain microvascular endothelial cells (mBMEC) were prepared using a modified protocol already described^44–46^. Briefly, brains were collected from four to six-week-old C57BL/6 mice, minced in Hanks balanced salt solution (HBSS; Life Science Technology) and homogenized gently in a Dounce type homogenizer. Myelin was removed by 18% Dextran suspension (Dextran 60–90,000; USB, Cleveland, OH) and centrifuging at 8000 rpm for 10 min at 4 °C. Red blood cells were removed by centrifuging isolated microvessels in a Percoll gradient (Thermo Fisher Scientific) at 2700 rpm for 11 min. The isolated microvessels were digested in HBSS solution containing 1 μg/ml collagenase/dispase (Roche, Indianapolis, IN), 10 U/ml DNAase I (Sigma-Aldrich, St Louis, MO) and 1 µg/ml Na-p-tosyl-L-lysine chloromethyl ketone (TLCK) for 20 min at 37 °C and precipitated with CD31 coated magnet beads (Dynabeads, Life Science technology). These vessels were further cultured in Dulbecco’s Modified Eagles medium (DMEM, Invitrogen, Carlsbad, CA) supplemented with 10% inactivated fetal bovine serum (FBS), 2.5 µg/ml heparin (Sigma-Aldrich, St Louis, MO), 20 mM HEPES, 2 mM glutamine, 1X antibiotic/antimycotic (all from Invitrogen, Carlsbad, CA), endothelial cell growth supplement (BD Bioscience, San Jose, CA) and grown in 6 well plates coated with collagen type IV (BD Bioscience, San Jose, CA). This protocol typically produces primary endothelial cell cultures that are approximately 99% pure (as determined by immunocytochemistry with an anti-PECAM-1 antibody; BD Bioscience, San Jose, CA).

Mouse brain endothelial cell lines (mBECL) were purchased from Angio-Proteomie (Boston MA) and cultured in medium (DMEM supplemented with 10% fetal bovine serum, 2 mM glutamine and 1X antibiotic/antimycotic; Invitrogen, Carlsbad, CA) at 37 °C in a humidified incubator (10%CO_2_/90% air).

Expression of PRX protein in mouse endothelial cells was performed by infection with recombinant lentiviruses, as in the prior section using polybrene. We used two controls for functional studies of PRX lentivirus infected mouse endothelial cells: (1) cells were infected with RFP lentivirus at the same multiplicity of infection and (2) cells were treated with polybrene without virus. In all control studies, the RFP virus had no effect compared to non-infected mouse endothelial cells. Therefore, to simplify the presentation of data, we only show uninfected cells as controls.

### Permeability coefficient (PC) assessments

The *in vitro* permeability of mBMEC monolayers was measured as described in Kazakoff *et al*.^47^, and modified in our laboratory^44–46^. The cells were seeded on the collagen IV coat insert in a Transwell dual chamber system at a density of 10,000 cells per square cm which allows the formation of cell barriers 1–7 day after initial plating.

The permeability coefficient (PC; cm/min) of monolayers was calculated for the tracers FITC-inulin (5 kDa) or dextran-Texas red 20 kDa and 40 kDa (1 μg/ml, Sigma-Aldrich) at different time points 1–7 days after initial platting. FITC-inulin and dextran-Texas Red concentrations in lower and upper chambers were evaluated on a fluorescent reader (Infinity FL200) using following formula:

1 $$PC=\frac{[C(B)T-C(B)]\cdot V(B)\cdot 2}{[C(A)+C(A)T]\cdot A\cdot T}$$

where C(B) and C(B)T are the concentrations (ng/ml) of tracer in the basal (receiving) chamber at the start and at the end of the time interval, respectively, and V(B) is the volume of the basal chamber (in ml). C(A) and C(A)T are, respectively, the concentrations of tracer in the apical (donor) chamber at the start and at the end of the time interval and (C(A) + C(A)T)/2 is the average concentration over the time interval. T is the duration of the time interval (in hours), while A is the area of the filter (in square cm). All samples were read on fluorescent reader (Bio-Tek Instruments, Inc., Winooski, VT U.S.A.; emission 485 and excitation 540 nm). The concentration of FITC-albumin in samples was calculated from a standard curve derived using known concentrations of tracer. Both uninfected cells and RFP lentivirus infected controls were studied and these groups showed identical results. Only data on uninfected controls are shown.

### Transendothelial electrical resistance (TEER) measurements

TEERs were measured in Endohm™ TEER measurement chambers equipped with an EVOM resistance meter (World Precision Instruments, Sarasota, FL). All experiments were carried out in triplicate in three independent experiments. Both uninfected cells and RFP lentivirus infected controls were studied and these groups showed identical results. Only data on uninfected controls are shown.

### Cell transfection and immunofluorescence

Mouse brain endothelial cell lines (mBECL) were grown to ~70% confluence and then transfected with GFP fusion clones L-PRX, S-PRX and D-PRX using Lipofectamine (Life Technologies) in Opti-MEM medium (Invitrogen) for 6 hrs according to the manufacturer’s instructions. After 24 hrs the samples examined for PRX expression and localization.

For immunofluorescence staining, cell samples were preincubated in blocking solution containing 5% normal horse serum and 0.05% Triton 100X (Sigma Aldrich, MO) in PBS for 30 min and then incubated overnight at 4 °C with the following antibodies: anti-claudin-5 and anti-ZO-1–Alexa Flour 594 conjugated (Life Science Technology). Reactions were visualized by Texas red anti-mouse antibodies. All samples were viewed on a confocal laser scanning microscope (Nikon).

### Statistics

Unpaired Student’s t-test and one-way analysis of variance (ANOVA), with Bonferroni and Tukey’s post hoc tests were used to test group level differences (Prism analysis software). A probability value <0.05 was regarded as statistically significant.

## Electronic supplementary material

Supplementary info

## References

[1] Hawkins BT, Davis TP (2005). The blood-brain barrier/neurovascular unit in health and disease. Pharmacological reviews.

[2] Obermeier B, Daneman R, Ransohoff RM (2013). Development, maintenance and disruption of the blood-brain barrier. Nature medicine.

[3] Zlokovic BV (2008). The blood-brain barrier in health and chronic neurodegenerative disorders. Neuron.

[4] Stamatovic SM, Johnson AM, Keep RF, Andjelkovic AV (2016). Junctional proteins of the blood-brain barrier: New insights into function and dysfunction. Tissue barriers.

[5] Krause G (2008). Structure and function of claudins. Biochimica et biophysica acta.

[6] Furuse M (1993). Occludin: a novel integral membrane protein localizing at tight junctions. The Journal of cell biology.

[7] Martin-Padura I (1998). Junctional adhesion molecule, a novel member of the immunoglobulin superfamily that distributes at intercellular junctions and modulates monocyte transmigration. The Journal of cell biology.

[8] Stevenson BR, Siliciano JD, Mooseker MS, Goodenough DA (1986). Identification of ZO-1: a high molecular weight polypeptide associated with the tight junction (zonula occludens) in a variety of epithelia. The Journal of cell biology.

[9] Jesaitis LA, Goodenough DA (1994). Molecular characterization and tissue distribution of ZO-2, a tight junction protein homologous to ZO-1 and the Drosophila discs-large tumor suppressor protein. The Journal of cell biology.

[10] Haskins J, Gu L, Wittchen ES, Hibbard J, Stevenson BR (1998). ZO-3, a novel member of the MAGUK protein family found at the tight junction, interacts with ZO-1 and occludin. The Journal of cell biology.

[11] Citi S, Sabanay H, Jakes R, Geiger B, Kendrick-Jones J (1988). Cingulin, a new peripheral component of tight junctions. Nature.

[12] Yamamoto T (1997). The Ras target AF-6 interacts with ZO-1 and serves as a peripheral component of tight junctions in epithelial cells. The Journal of cell biology.

[13] Zhong Y (1993). Monoclonal antibody 7H6 reacts with a novel tight junction-associated protein distinct from ZO-1, cingulin and ZO-2. The Journal of cell biology.

[14] Beatch M, Jesaitis LA, Gallin WJ, Goodenough DA, Stevenson BR (1996). The tight junction protein ZO-2 contains three PDZ (PSD-95/Discs-Large/ZO-1) domains and an alternatively spliced region. The Journal of biological chemistry.

[15] Gonzalez-Mariscal L, Betanzos A, Avila-Flores A (2000). MAGUK proteins: structure and role in the tight junction. Seminars in cell & developmental biology.

[16] Itoh M (1999). Direct binding of three tight junction-associated MAGUKs, ZO-1, ZO-2, and ZO-3, with the COOH termini of claudins. The Journal of cell biology.

[17] Van Itallie CM, Tietgens AJ, Krystofiak E, Kachar B, Anderson JM (2015). A complex of ZO-1 and the BAR-domain protein TOCA-1 regulates actin assembly at the tight junction. Molecular biology of the cell.

[18] Fanning AS (2007). The unique-5 and -6 motifs of ZO-1 regulate tight junction strand localization and scaffolding properties. Molecular biology of the cell.

[19] Utepbergenov DI, Fanning AS, Anderson JM (2006). Dimerization of the scaffolding protein ZO-1 through the second PDZ domain. The Journal of biological chemistry.

[20] Lee SJ (2017). Large-scale identification of human cerebrovascular proteins: Inter-tissue and intracerebral vascular protein diversity. Plos One.

[21] Dytrych L, Sherman DL, Gillespie CS, Brophy PJ (1998). Two PDZ domain proteins encoded by the murine periaxin gene are the result of alternative intron retention and are differentially targeted in Schwann cells. The Journal of biological chemistry.

[22] Uhlen, M. *et al*. A human protein atlas for normal and cancer tissues based on antibody proteomics. *Molecular & cellular proteomics: MCP* **4**, 1920–1932. 10.1074/mcp.M500279-MCP200 16127175

[23] Uchida Y, Terasaki T (2011). Quantitative targeted absolute proteomics-based ADME research as a new path to drug discovery and development: methodology, advantages, strategy, and prospects. Journal of pharmaceutical sciences.

[24] Uchida Y (2011). Quantitative targeted absolute proteomics of human blood-brain barrier transporters and receptors. J Neurochem.

[25] Alanne MH (2009). Tight junction proteins in human Schwann cell autotypic junctions. The journal of histochemistry and cytochemistry: official journal of the Histochemistry Society.

[26] Maddala R (2011). Periaxin is required for hexagonal geometry and membrane organization of mature lens fibers. Developmental biology.

[27] Gillespie CS, Sherman DL, Blair GE, Brophy PJ (1994). Periaxin, a novel protein of myelinating Schwann cells with a possible role in axonal ensheathment. Neuron.

[28] Gillespie CS (2000). Peripheral demyelination and neuropathic pain behavior in periaxin-deficient mice. Neuron.

[29] Boerkoel CF (2001). Periaxin mutations cause recessive Dejerine-Sottas neuropathy. American journal of human genetics.

[30] Boerkoel CF (2002). Charcot-Marie-Tooth disease and related neuropathies: mutation distribution and genotype-phenotype correlation. Annals of neurology.

[31] Guilbot A (2001). A mutation in periaxin is responsible for CMT4F, an autosomal recessive form of Charcot-Marie-Tooth disease. Human molecular genetics.

[32] Sherman DL, Wu LM, Grove M, Gillespie CS, Brophy PJ (2012). Drp2 and periaxin form Cajal bands with dystroglycan but have distinct roles in Schwann cell growth. The Journal of neuroscience: the official journal of the Society for Neuroscience.

[33] Shi Y, Zhang L, Yang T (2014). Nuclear export of L-periaxin, mediated by its nuclear export signal in the PDZ domain. Plos one.

[34] Sherman DL, Brophy PJ (2000). A tripartite nuclear localization signal in the PDZ-domain protein L-periaxin. The Journal of biological chemistry.

[35] Galicich JH, French LA, Melby JC (1961). Use of dexamethasone in treatment of cerebral edema associated with brain tumors. The Journal-lancet.

[36] Ingraham FD, Matson DD, Mc LR (1952). Cortisone and ACTH as an adjunct to the surgery of craniopharyngiomas. The New England journal of medicine.

[37] Tsukamoto T, Nigam SK (1999). Role of tyrosine phosphorylation in the reassembly of occludin and other tight junction proteins. The American journal of physiology.

[38] Xu J (2008). Early embryonic lethality of mice lacking ZO-2, but Not ZO-3, reveals critical and nonredundant roles for individual zonula occludens proteins in mammalian development. Molecular and cellular biology.

[39] Dong H, Blaivas M, Wang MM (2012). Bidirectional encroachment of collagen into the tunica media in cerebral autosomal dominant arteriopathy with subcortical infarcts and leukoencephalopathy. Brain Res.

[40] Zhang X, Lee SJ, Young MF, Wang MM (2015). The small leucine-rich proteoglycan BGN accumulates in CADASIL and binds to NOTCH3. Translational stroke research.

[41] Roberts LM (2008). Subcellular localization of transporters along the rat blood-brain barrier and blood-cerebral-spinal fluid barrier by *in vivo* biotinylation. Neuroscience.

[42] Lee SJ, Meng H, Elmadhoun O, Blaivas M, Wang MM (2011). Cerebral Autosomal Dominant Arteriopathy With Subcortical Infarcts and Leukoencephalopathy Affecting an African American Man: Identification of a Novel 15-Base Pair NOTCH3 Duplication. Arch Neurol.

[43] Meng, H., Zhang, X., Lee, S. J. & Wang, M. M. Von Willebrand factor inhibits mature smooth muscle gene expression through impairment of Notch signaling. *Plos One* **8** (2013). 10.1371/journal.pone.0075808 PMC3781053 24086636

[44] Dimitrijevic OB, Stamatovic SM, Keep RF, Andjelkovic AV (2006). Effects of the chemokine CCL2 on blood-brain barrier permeability during ischemia-reperfusion injury. Journal of cerebral blood flow and metabolism: official journal of the International Society of Cerebral Blood Flow and Metabolism.

[45] Stamatovic SM, Keep RF, Wang MM, Jankovic I, Andjelkovic AV (2009). Caveolae-mediated internalization of occludin and claudin-5 during CCL2-induced tight junction remodeling in brain endothelial cells. The Journal of biological chemistry.

[46] Stamatovic SM, Sladojevic N, Keep RF, Andjelkovic AV (2012). Relocalization of junctional adhesion molecule A during inflammatory stimulation of brain endothelial cells. Molecular and cellular biology.

[47] Kazakoff PW, McGuire TR, Hoie EB, Cano M, Iversen PL (1995). An *in vitro* model for endothelial permeability: assessment of monolayer integrity. In vitro cellular & developmental biology. Animal.

