# Supplementary material for: Expression of periaxin (PRX) specifically in the human cerebrovascular system: PDZ domain-mediated strengthening of endothelial barrier function
Source: Sci Rep. 2018 Jul 3;8:10042. doi: 10.1038/s41598-018-28190-7 (PMC6030167; doi:10.1038/s41598-018-28190-7)
Supplement: Supplementary file 1 — Supplementary info [file 41598_2018_28190_MOESM1_ESM.pdf]

Expression of periaxin (PRX) specifically in the human cerebrovascular system: PDZ domain-mediated strengthening of endothelial barrier function

SUPPLEMENTAL INFORMATION

Michael M. Wang[1-4 *], Xiaojie Zhang[1,2], Soo Jung Lee[1,2], Snehaa Maripudi[1,3], Richard F. Keep[2,4,5], Allison M. Johnson[4,6,7], Svetlana M. Stamatovic[6,7], and Anuska V. Andjelkovic[4,6,7]

From the [1]Departments of Neurology, [2]Molecular and Integrative Physiology, [5]Neurosurgery, and [7]Pathology, University of Michigan, Ann Arbor, MI 48109; [3]Neurology Service, VA Ann Arbor Healthcare System, Department of Veterans Affairs, Ann Arbor, MI 48105; [4]Neuroscience and [6]Molecular and Cellular Pathology Graduate Programs, University of Michigan, Ann Arbor, MI 48109

Running Title: Human-specific endothelial barrier enhancer

Address Correspondence to: Michael M. Wang, 7725 Medical Science Building II Box 5622, 1137 Catherine St., Ann Arbor, MI 48109-5622, Tel. 734-763-5453; Fax 734-936-8813; E-Mail: micwang@umich.edu

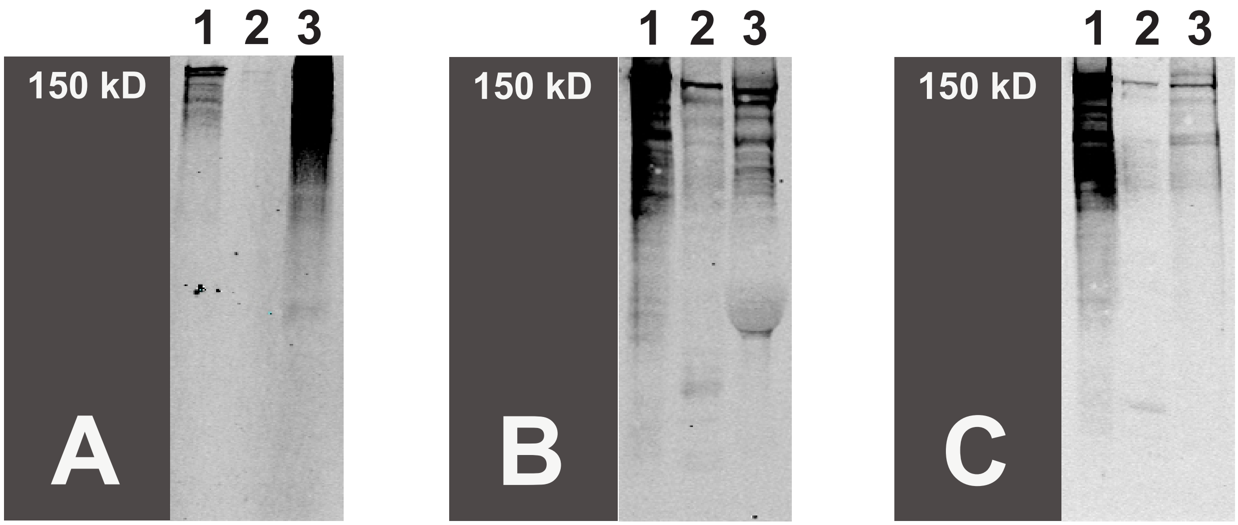

Supplemental Figure 1. Western blot analysis of PRX in human whole brain and human brain vessel fractions. Three brain samples were analyzed for PRX expression, shown in the three independent Western blots. In each blot, Lane 1 contains protein lysates from 293 cells transfected with L-PRX. This was deliberately underloaded to enable visualization of individual bands. Lane 2 shows human whole brain (Hwb) protein lysates; Lane 3 shows vessel fractions from the same brain. An overexposed version of Figure 3 is shown in (A; 10% gel). (B) and (C) include proteins from two independent brains. Note that the levels of tubulin in the Hwb were much lower than in vessels in (B) and (C) (8% gel). These blots show that PRX is found in multiple bands from protein fractions from both brain and transfected 293 cells. The main band in transfected cells is slightly higher than the main band from brain. But, there appeared to be overlap in the bands generated from transfeccted 293 and brain expressed proteins.

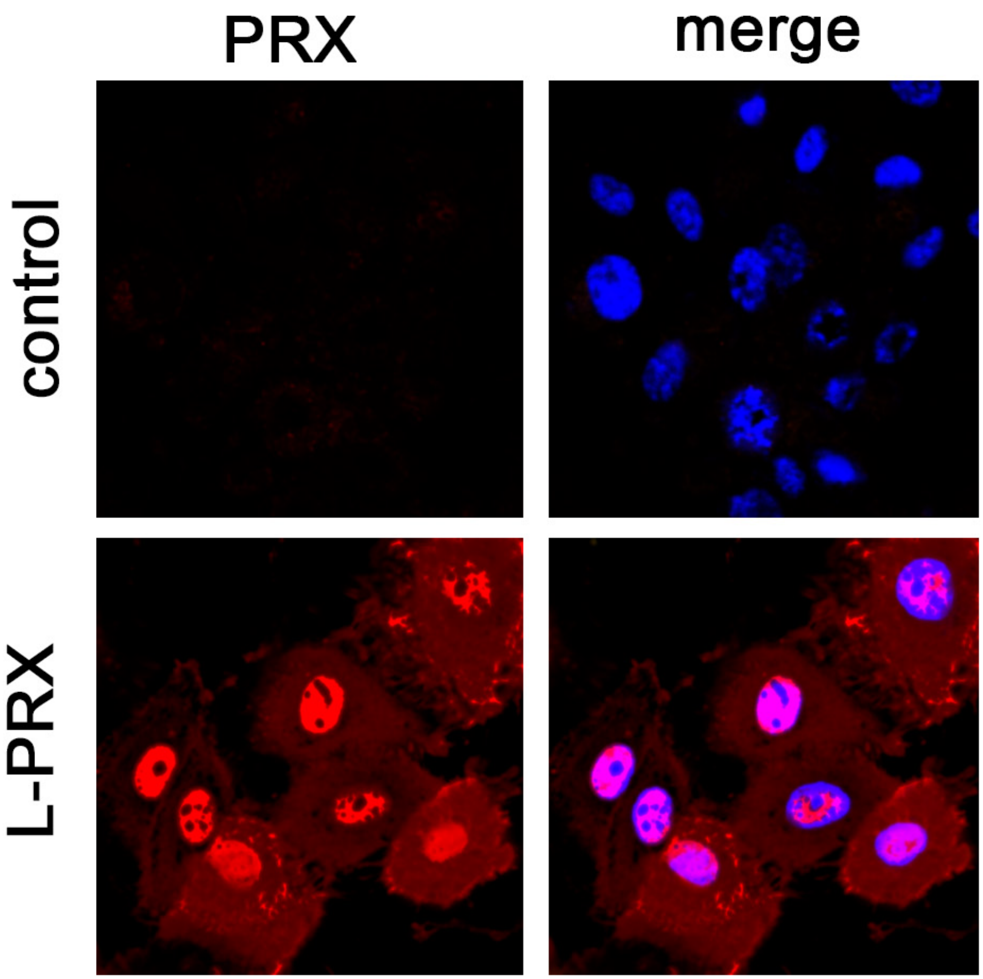

Supplemental Figure 2. L-PRX protein localization in cerebral endothelial cells. Cultured murine brain endothelial cells were transfected with empty control or L-PRX expression vector. L-PRX was not tagged. Cells were then immunostained for PRX and counterstained with DAPI. Confocal images demonstrate predominantly nuclear localization of L-PRX.

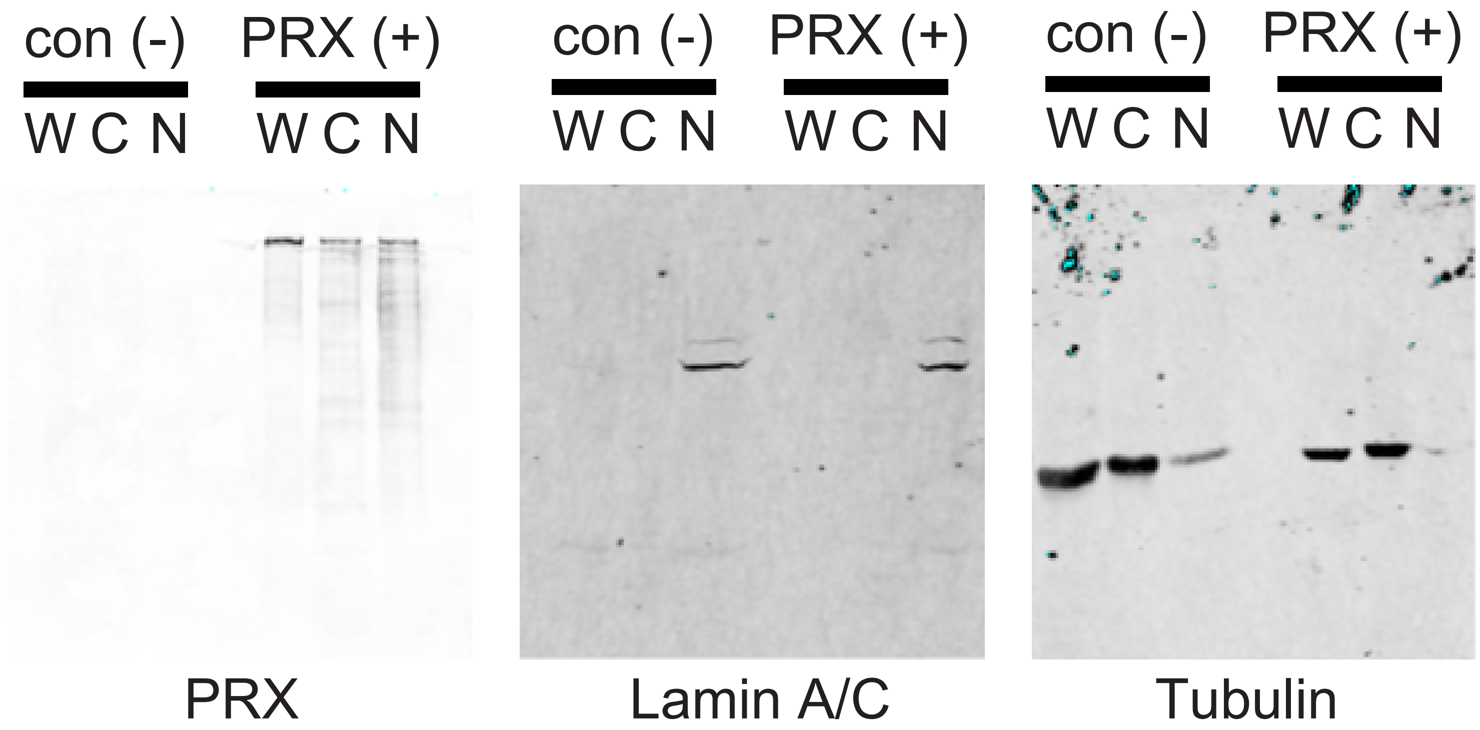

Supplemental Figure 3. L-PRX protein localization in cultured cells. Culture 293 cells (control (-)) and PRX-transfected cell (+) were separated into nuclear and cytoplasmic fractions and immunoblotted for PRX, Lamin A/C (a nuclear marker) or tubulin (enriched in cytosol). Whole cell lysates (W), cytoplasmic proteins (C), and nuclear proteins (N) were immunoblotted. The results suggest that a significant amount of PRX is present in the nucleus, which has a comparable amount of PRX compared to the cytoplasm, though there is a significantly smaller level of tubulin in the nuclear fraction.

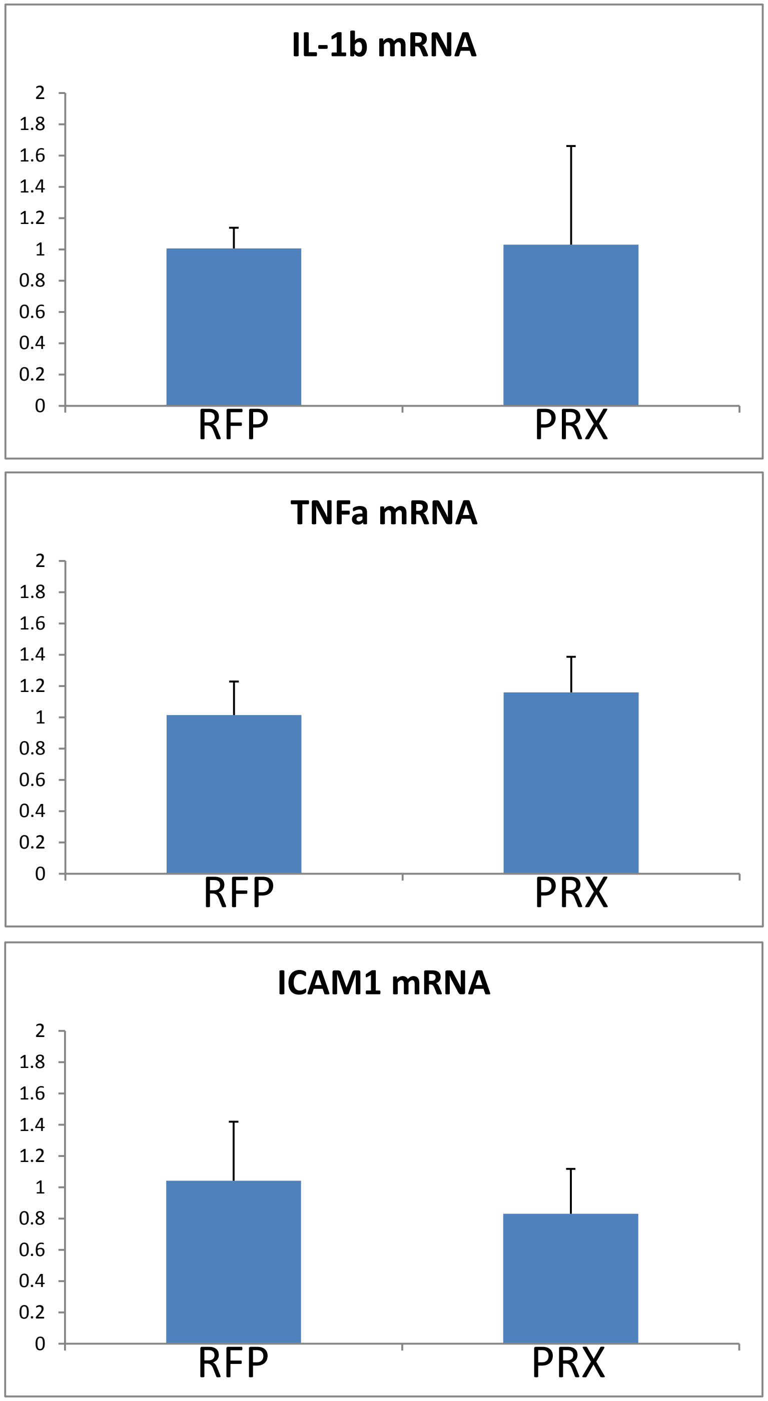

Supplemental Figure 4. L-PRX effects on expression of IL-1b, TNFa, and ICAM1 mRNA. . Cultured human brain endothelial cells RFP control of L-PRX expression vector. mRNA from these cells was analyzed by quantitative RT-PCR for markers shown. There were no changes seen between any of the groups for these markers.
